# Targeting DNAJB9, a novel ER luminal co-chaperone, to rescue ΔF508-CFTR

**DOI:** 10.1038/s41598-019-46161-4

**Published:** 2019-07-08

**Authors:** Yunjie Huang, Kavisha Arora, Kyu Shik Mun, Fanmuyi Yang, ChangSuk Moon, Sunitha Yarlagadda, Anil Jegga, Timothy Weaver, Anjaparavanda P. Naren

**Affiliations:** 1 Division of Pulmonary Medicine, Cincinnati Children’s Hospital Medical Center, Cincinnati, OH 45229 United States; 2 Division of Biomedical Informatics, Cincinnati Children’s Hospital Medical Center, Cincinnati, OH 45229 United States; 3 Division of Pulmonary Biology, Cincinnati Children’s Hospital Medical Center, Cincinnati, OH 45229 United States

**Keywords:** ER-associated degradation, Mechanisms of disease

## Abstract

The molecular mechanism of Endoplasmic Reticulum-associated degradation (ERAD) of Cystic fibrosis transmembrane-conductance regulator (CFTR) is largely unknown. Particularly, it is unknown what ER luminal factor(s) are involved in ERAD. Herein, we used ProtoArray to identify an ER luminal co-chaperone, DNAJB9, which can directly interact with CFTR. For both WT- and ΔF508 (deletion of phenylalanine at position 508, the most common CF-causing mutant)-CFTR, knockdown of DNAJB9 by siRNA increased their expression levels on the cell surface and, consequently, upregulated their function. Furthermore, genetic ablation of DNAJB9 in WT mice increased CFTR expression and enhanced CFTR-dependent fluid secretion in enteroids. Importantly, DNAJB9 deficiency upregulated enteroids’ fluid secretion in CF mice (homozygous for ΔF508), and silencing one allele of DNAJB9 is sufficient to rescue ΔF508-CFTR *in vitro* and *in vivo*, suggesting that DNAJB9 may be a rate-limiting factor in CFTR ERAD pathway. Our studies identified the first ER luminal co-chaperone involved in CFTR ERAD, and DNAJB9 could be a novel therapeutic target for CF.

## Introduction

Endoplasmic Reticulum (ER)-associated degradation (ERAD) system plays an important role in protein homeostasis. ERAD mediates degradation of terminally misfolded or unassembled proteins via multiple steps: recognition, retrotranslocation, ubiquitination, and proteasomal degradation^1^. Molecular (co)chaperones from both cytosol and ER lumen including Hsp40, Hsp70, and Hsp90, play a critical role in protein folding and degradation^2^.

Cystic Fibrosis transmembrane-conductance regulator (CFTR) is a chloride/bicarbonate channel expressed on the apical membrane of epithelial cells. It is a complex transmembrane protein consisting of 1,480 amino acids and full assembly in the ER requires over 30 minutes^3^. CFTR has two membrane spanning domains (MSD), two nucleotide-binding domains (NBD) and one regulatory (R) domain. Because of its complex structure, in multiple commonly used cell lines, up to 70% of newly synthesized CFTR is not able to achieve an energetically favorable, native conformation to pass Quality Control (QC) and consequently is degraded prematurely^4,5^.

Mutations of CFTR cause one of the most common genetic diseases, Cystic Fibrosis^6^, which affects about one in 3,500 newborns in the United States^7^. Defective CFTR leads to dehydration of the epithelial surface, and consequently impairment of multiple organs, including lung, pancreas and intestine^8,9^. Largely, these mutations lead to protein misfolding and subsequent degradation by ERAD, resulting in loss of protein at the plasma membrane (PM). Among the mutations, deletion of phenylalanine at position 508 (ΔF508) is the most common mutation, present in ≥85% CF patients (2016 CF Patient Registry). ΔF508 affects not only the folding of NBD1 domain containing the mutation but also global conformation by interrupting domain-domain interactions^10–12^. Thus, almost all of ΔF508-CFTR is immediately recognized and degraded by ERAD^4,13^.

Cytosolic chaperones have been found to be involved in ERAD of ΔF508-CFTR both co- and post-translationally. Hsc70 mediates ERAD of CFTR at different stages by interacting with different sets of co-chaperones. It has been shown that Hsc70 along with DNAJB12 play an important role in ΔF508-CFTR degradation during translation^14^. In contrast, by partnering with CHIP, Hsc70 promotes ΔF508-CFTR degradation post-translationally^15^, a process that is regulated by co-chaperones, *i.e*. HspBP1^16^, BAG-2^17^ and Hdj2/DNAJA1^18^. Cytosolic chaperones, such as Hsp70 and Hsp90 appear to promote CFTR maturation & stabilization; however, prolonged binding to Hsp90 may target CFTR for degradation^19,20^. Interestingly, these ERAD pathways can be manipulated to rescue ΔF508-CFTR for surface and functional expression. For example, overexpressing BAG-2 has been shown to increase both immature and mature CFTR by inhibiting CHIP ubiquitin ligase activity^17^.

Besides the cytosolic chaperones, CFTR biogenesis and degradation is also regulated by the ER luminal environment, including molecular chaperones. Caplan *et al*. have argued that transiently inhibiting the ER-calcium pump would allow ΔF508-CFTR to exit ER for PM expression^21^, suggesting that ER luminal environment is important for CFTR retention. Of the chaperones involved in CFTR biosynthesis, FKBP8, a peptidylpropyl isomerase, has been shown to interact with, and stabilize both WT- and ΔF508-CFTR^22^. In addition, ERp29 was also identified to play a positive role in assisting surface expression of WT- and ΔF508-CFTR^23^. Surprisingly, although CFTR has two N-glycosylation sites, recent data suggests that the ER lectin system, including calnexin and calreticulin, appears not to be responsible for the ER retention of ΔF508-CFTR^24,25^, although glycosylation is important for CFTR stability post-translationally^25^. It has been noted previously that the most abundant ER chaperone, BiP, is not associated with CFTR^26–28^. Therefore, it remains unknown as to which luminal proteins are involved in the ERAD of CFTR. Additionally, it is not known if ΔF508-CFTR could be rescued by manipulating these chaperones.

In this study, we performed ProtoArray, a protein microarray exploring protein-protein interactions, to identify CFTR-interacting ER chaperones. DNAJB9 was found to have relatively high signal intensity to CFTR. DNAJB9 is also termed ERdj4; in order to avoid confusion, hereafter DNAJB9 will be used. *In vitro* experiments and animal model studies suggest that DNAJB9 plays an important role in mediating ERAD of both WT- and ΔF508-CFTR. Importantly, altering the CFTR-DNAJB9 interaction may be a novel strategy to rescue ΔF508-CFTR.

## Results

### Discovery of DNAJB9 as CFTR-interacting partner using ProtoArray

**Figure 1:**
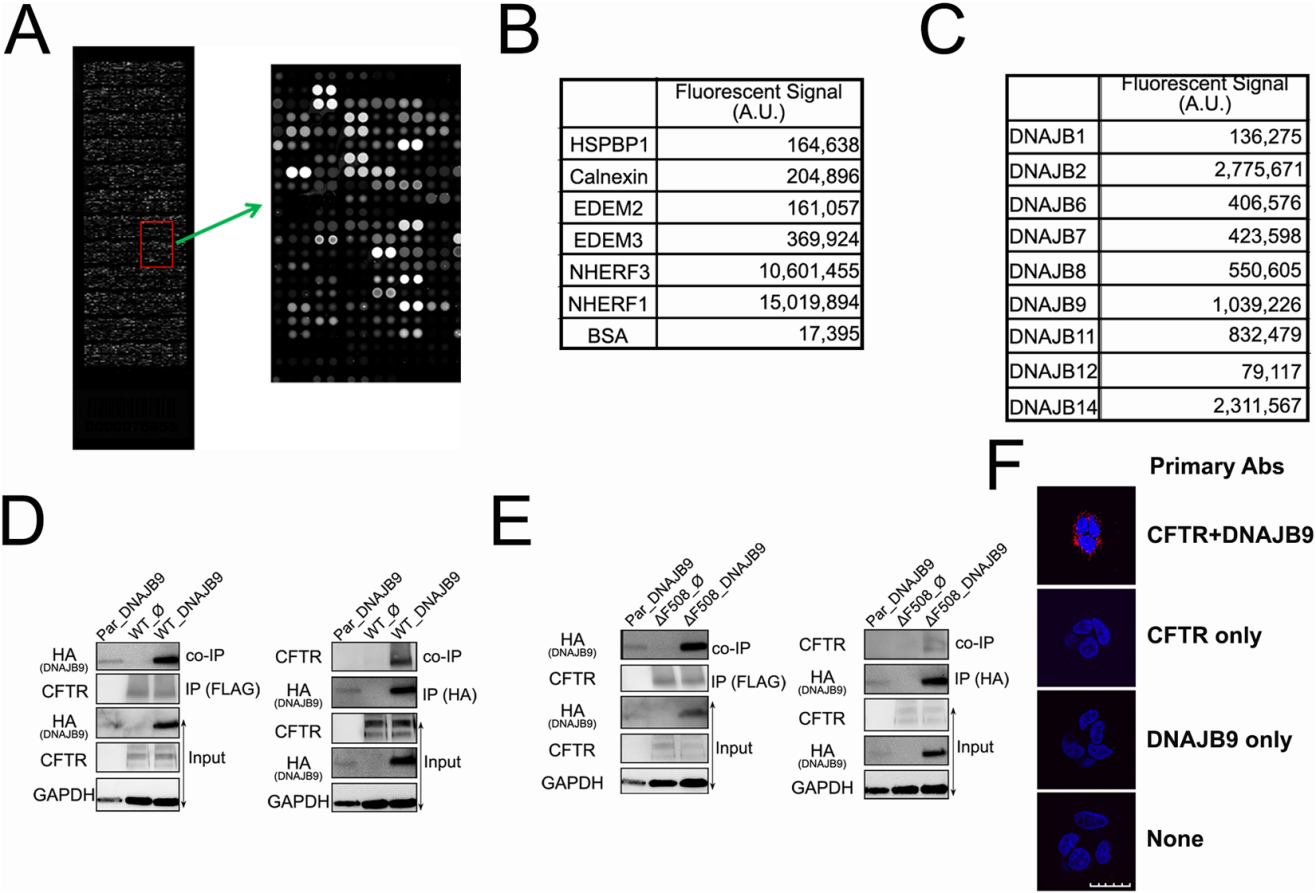
Direct interaction between CFTR and DNAJB9. **(A)** Illustration of ProtoArray Chip. The more CFTR captured, the brighter the spot, the higher signal intensity. **(B)** Known CFTR-interacting proteins were revealed by ProtoArray. **(C)** Different binding of DNAJB family members with CFTR was determined by protoArray. Data presented as mean of duplicate. **(D**,**E)** Western blotting of co-immunoprecipitation. HEK293 cell lines stably expressing WT- (D) and ΔF508- (E) CFTR were transiently transfected with empty vector or DNAJB9-3HA vector. Forty-eight hours after transfection, immunoprecipitation was performed using anti-FLAG antibody beads (Left) and anti-HA antibody beads (Right). Precipitate and total cell lysate (input) were then subjected to western Blotting using anti-CFTR, anti-HA and anti-GAPDH antibodies. Data were representative of three independent experiments. Full-length blots are presented in Fig. S9. **(F)** Proximity Ligation Assay (PLA) using T84 cells. PLA assay was performed according to the manufacturer’s protocol using T84 cells which are known to endogenously express CFTR. From top to bottom, samples were treated with antibodies against both CFTR and DNAJB9, CFTR only, DNAJB9 only, and no primary ab. The interaction between CFTR and DNAJB9 was shown by red fluorescent; blue is DAPI staining. Data was representative of three independent experiments. Scale bar represents 20 µm.

Direct protein-protein interactions often have meaningful functions. ProtoArray technology (Invitrogen) has proven to be invaluable in identifying unelucidated protein-protein interactions in a variety of human studies^29–31^. The rationale for using ProtoArray for this study was to discover CFTR-interacting ER luminal proteins. To study QC mechanisms, we captured interacting partners on the microarray using purified full length FLAG-CFTR from mammalian HEK293 cells (Fig. S1). The bound CFTR was probed using mouse anti-FLAG monoclonal antibody followed by Alexa-488 conjugated secondary antibody and quantitation by a fluorescence microarray scanner. A stronger interaction results in a higher signal (Fig. 1A–C).

Using the microarray, over 2,000 interacting partners were identified, and distributed in various subcellular localizations, including cytoplasm, nucleus, extracellular, PM and others. The results were validated by determining the known CFTR-interacting proteins from different compartments; Na^+^/H^+^ Exchange Regulator Factor 1 (NHERF1, also known as EBP50) and NHERF3 (also known as PDZK1) are the scaffold proteins expressed in epithelial cell apical domains and can directly interact with the C-terminal of CFTR via their PDZ domains^32,33^. Therefore, as expected, NHERF1 and 3 showed strong signal in microarray studies (Fig. 1B). In contrast, proteins typically identified in CFTR co-immunoprecipitation studies, *e.g*. chaperone protein calnexin [CNX,^25,34^], co-chaperone HSP70 Binding Protein 1 [HspBP1,^16^] and ER degradation-enhancing α-mannosidase-like protein [EDEM,^25,34^] show much lower signal strength with CFTR (Fig. 1B), suggesting that ProtoArray has distinct ability to identify direct protein-protein interactions.

Multiple ER luminal chaperones bound CFTR with comparable signal intensity to known CFTR interactors; in particular, DNAJB9, a DNAJ (Hsp40) homology subfamily B member 9, bound CFTR with the relatively high signal intensity (Fig. 1C). Although the signal intensity for DNAJB9-CFTR is about 10-fold less than for NHERFs, it is 3–7 fold higher compared to other known proteins in Fig. 1B. DNAJB9 is a soluble ER luminal co-chaperone^35^, identified as inducible Heat Shock Protein upon cell stress^36^, and plays an important role in ERAD of misfolded proteins^36,37^. Other DNAJB family members also bound CFTR (Fig. 1C). Among them, DNAJB1, 6, 7, and 8 are cytosolic proteins^38–41^, while DNAJB2, DNAJB9, DNAJB11/ERjd3, DNAJB12 and DNAJB14 are ER proteins. Specifically, DNAJB2 is mainly expressed in neuronal cells^42^. DNAJB12 and DNAJB14 was demonstrated to be on the ER membrane with their functional J-domain facing the cytosol^41,43^. Although DNAJB11 is an ER luminal protein, it was suggested to assist BiP-mediated protein folding^44,45^. Given that newly synthesized CFTR is not associated with BiP^26^, we focused on DNAJB9, and wanted to test if the protein plays a role in ERQC for CFTR.

### DNAJB9 is associated with WT- and ΔF508-CFTR

To validate the interaction between CFTR and DNAJB9 in cells, co-immunoprecipitation (co-IP) was performed using HEK293 cell lines that stably express WT- and ΔF508-CFTR. Parental HEK293 cells (Par) were also recruited as specificity control. DNAJB9-HA^36^ was transiently overexpressed in these cells. Immunoprecipitation of FLAG-CFTR recovered a fraction of DNAJB9 from cells stably expressing WT-CFTR, while in DNAJB9 immuneprecipitates using anti-HA antibody, WT-CFTR was present as well (Fig. 1D). Although ΔF508 alters CFTR global conformation^46^, the structure within ER lumen has not been elucidated. We tested whether DNAJB9 was associated with ΔF508-CFTR. As expected, a fraction of DNAJB9 was present in the immuneprecipitates of ΔF508-CFTR (Fig. 1E). Conversely, in DNAJB9-HA immunoprecipitates, a small fraction of ΔF508-CFTR was able to be detected as well (Fig. 1E). In contrast, Par control shown no CFTR or little DNAJB9 in co-IP, although it was noticed that DNAJB9 expression in Par control is much lower which is probably due to low transfection efficiency. To exclude the possibility that interaction between CFTR and DNAJB9 seen in the co-IP experiments was random association between these two proteins during lysis and incubation, chemical crosslinking was performed using DSP crosslinker before co-IP and the immunoprecipitates were subjected to immunoblotting using WES system (ProteinSimple Inc.). As shown in Fig. S2A,B, in both WT- and ΔF508-CFTR immuneprecipitates, a small fraction of DNAJB9-HA was detected. The interaction between CFTR and DNAJB9 was further validated by looking at their endogenous association using proximity ligation assay (PLA) in T84 cells. As shown in Fig. 1F, PLA signal was detected only when antibodies against both CFTR and DNAJB9 were used. Together with ProtoArray data, these results suggest that DNAJB9 is very likely a direct, interacting partner of both WT-and ΔF508-CFTR.

### DNAJB9 promotes turnover of CFTR

DNAJB9 is a soluble ER-localized Type II DnaJ homologue, containing N-terminal J domain and the Gly/Phe-rich domain, and C-terminal substrate binding domain^47^. Initially DNAJB9 was identified as an ER stress inducible co-chaperone with a role in protecting cells from ER stress^48^. Consistently, subsequent studies suggested that the general function for DNAJB9 was to mediate ERAD of misfolded proteins, *e.g*. surfactant protein C^36^ and ENaC^49^; knockdown of DNAJB9 by siRNA increased the target proteins’ stability^36^. Several lines of evidence have suggested that DNAJB9 is able to couple the substrate binding and association with ERAD machinery in a BiP-independent manner^35–37^. Given the critical roles of DNAJB9 in maintaining protein homeostasis, DNAJB9-deficient mice, although viable, showed constitutive ER stress^50^. Based on these findings and the potential direct interaction between CFTR and DNAJB9, we hypothesized that DNAJB9 acts as a functional component in CFTR ERAD pathway.

**Figure 2:**
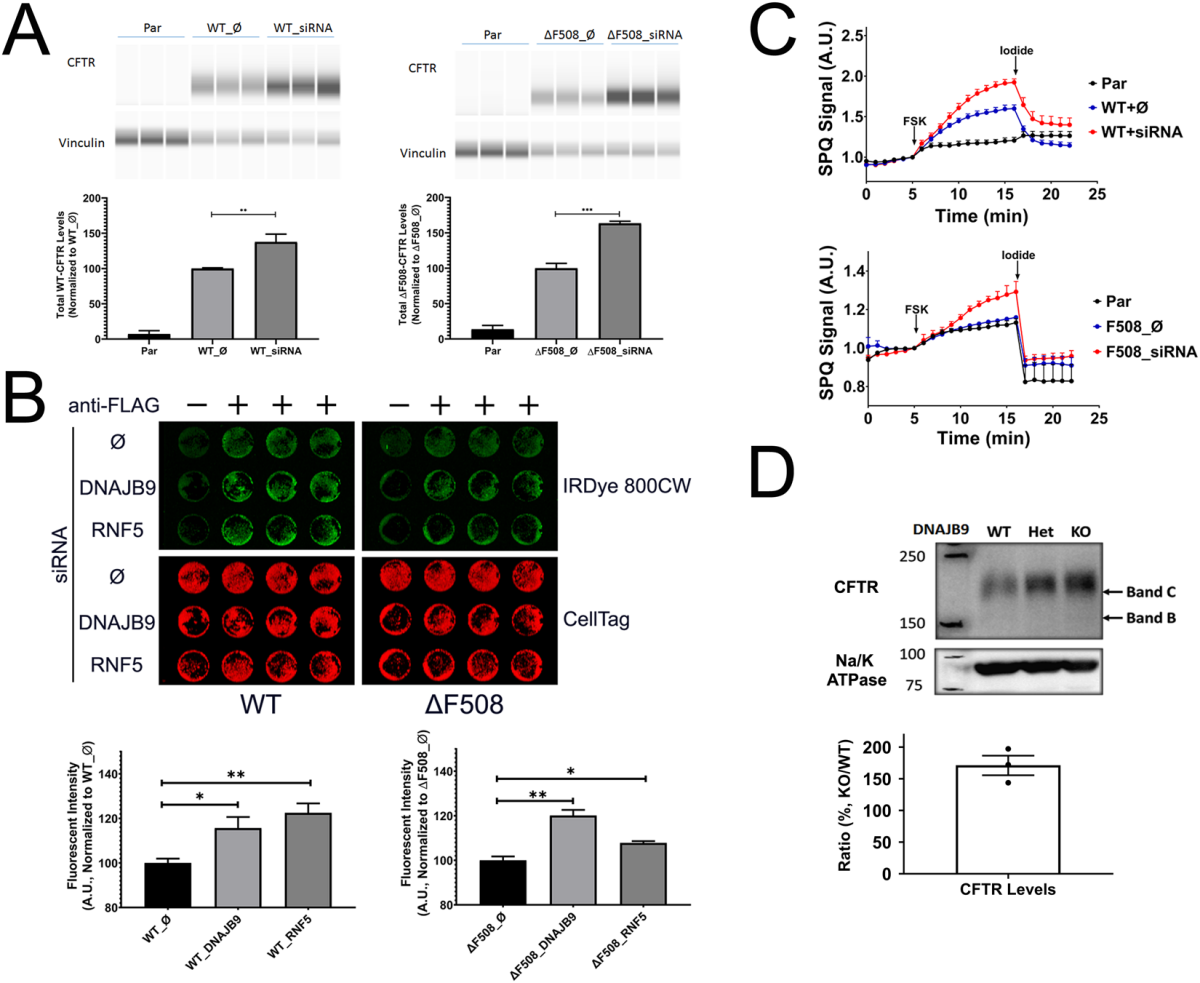
Knockdown DNAJB9 rescues both WT- and ΔF508-CFTR. **(A–C)** Knockdown of DNAJB9 by siRNA increased CFTR surface and functional expression in HEK293 cells. HEK293 cell lines stably expressing WT- or ΔF508-CFTR were transiently transfected with empty vector (Ø) or siRNA against DNAJB9 (siRNA) for 36–48 hours before assays. Parental cells that do not express CFTR were recruited as a negative control. All data were representative of three independent experiments. **(A)** WES analysis of CFTR and Vinculin (loading control). Total cell lysate was analyzed by WES system using anti-CFTR and anti-Vinculin antibodies. Each sample was analysed in triplicate. Student’s *t*-test was performed to determine the statistical significance. (**P < 0.01; ***P < 0.005). **(B)** In-Cell western to determine surface CFTR levels. Cells were reverse transfected by siRNA against DNAJB9 and RNF5 in 96-well plate. Forty-eight hours after transfection, cells were then fixed and surface CFTR was probed by anti-FLAG antibody followed by IRDye 800CW conjugated Goat anti-Rabbit 2^nd^ antibody. CellTag 700 was used as cell quantity control. Samples without anti-FLAG antibody incubation were used as background control. Representative images were shown on the top and quantification was shown at the bottom. Each condition was done in triplicates. Student’s *t*-test was performed to determine the statistical significance. (*P < 0.05; **P < 0.01). **(C)** SPQ assay to assess CFTR function. Cells were seeded into 96-well plate 24 hours after transfection and then subjected to SPQ assay described in “Materials and Methods” section. A representative graph is shown for WT-CFTR (top) and ΔF508-CFTR (bottom). Each condition was performed in triplicate. **(D)** Knockdown DNAJB9 increased CFTR expression. Immunoblotting of mouse Jejunum membrane fraction from different genotypes, DNAJB9^+/+^ (WT), DNAJB9^+/−^ (Het), and DNAJB9^−/−^ (KO). CFTR and Na/K ATPase was probed using anti-CFTR and anti-Na/K ATPase antibodies, and Na/K ATPase was served as a membrane marker and loading control. Protein levels was quantified and ratio of KO/WT was determined. Full-length blots are presented in Fig. S11.

In previous studies, knockdown of functional ERAD components *e.g*. co-chaperone DNAJB12^14^ or E3 ligase RNF5^51^, increased total CFTR levels. Therefore, we asked if downregulation of DNAJB9 would lead to increased CFTR levels. Using WES system, compared to empty vector treated negative control, downregulation of DNAJB9 by siRNA increased total protein levels of WT- and ΔF508-CFTR (Fig. 2A).

Subsequently, we asked whether decreasing DNAJB9 expression would increase or rescue WT- and ΔF508-CFTR function on the surface, as several lines of evidence suggested that knockdown of a functional component of CFTR ERAD pathways would increase CFTR surface expression as well as its function^14,51^. In-Cell Western (ICW) was used to measure the surface levels of CFTR, and in parallel SPQ assay was performed to determine CFTR function. As expected, based on previous studies^51^, knockdown RNF5 by siRNA upregulated CFTR surface levels (Fig. 2B). Interestingly, knockdown of DNAJB9 by siRNA also increased surface expression of both forms of CFTR (Fig. 2B). Consistently, downregulation of DNAJB9 by siRNA enhanced CFTR function (Fig. 2C). It is noteworthy that knockdown of DNAJB9 using specific siRNA in our experimental settings could only lead to around 20–30% knockdown of DNAJB9 mRNA expression (Fig. S3), arguing that partially inhibiting DNAJB9 function can substantially change the folding kinetics of newly synthesized CFTR.

To further test the hypothesis that DNAJB9 plays a role in ERAD of CFTR, the ubiquitination levels of CFTR was examined upon DNAJB9 overexpression. As expected, overexpression DNAJB9 upregulated CFTR ubiquitination (Fig. S4); taken together, these results indicated that DNAJB9 is a functional component of the ERAD of CFTR and suggested that the DNAJB9-dependent ERAD pathway is a novel target for CF therapy.

### DNAJB9 deficiency rescued both WT- and ΔF508-CFTR in mice

**Figure 3:**
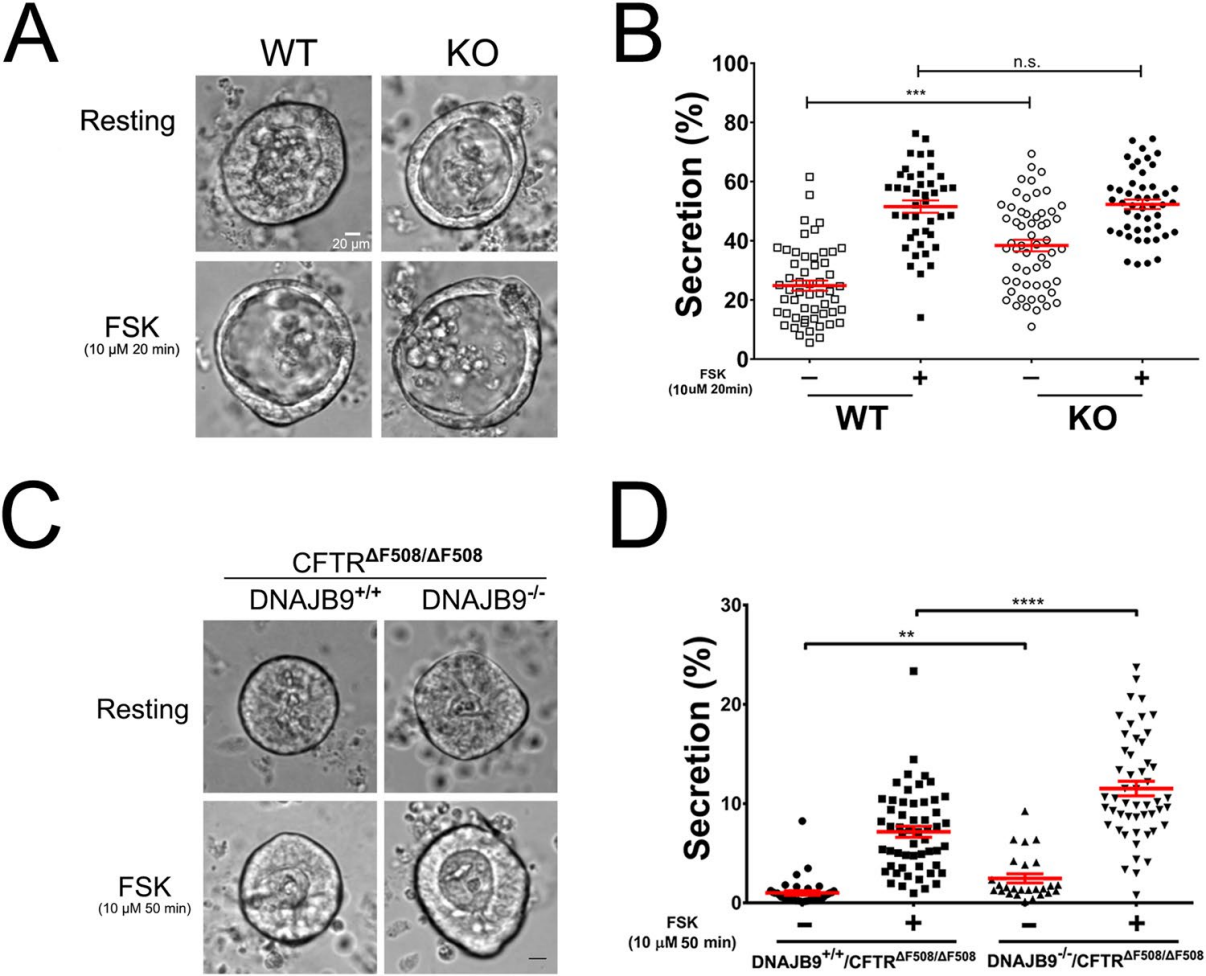
Genetic deficiency of DNAJB9 increased CFTR function in mouse intestinal organoids. Intestinal crypts from mouse in both WT-CFTR **(A,B)** and ΔF508-CFTR **(C,D)** background were isolated and cultured in matrigel at 37 °C to form intestinal organoids. Organoid fluid secretion as described in “Material and Method” section was performed in the absence or presence of FSK. **(A)** Representative organoid images, in both resting and FSK-stimulated states, were shown for comparison between WT and DNAJB9 KO mice. **(B)** Quantification of **(A)**. Each symbol represents one organoid. Data were representative of three independent experiments. **(C)** Representative organoid images, in both resting and FSK-stimulated states, were shown for comparison between DNAJB9^+/+^CFTR^ΔF508/ΔF508^ and DNAJB9^−/−^CFTR^ΔF508/ΔF508^ mice. **(B)** Quantification of **(C)**. Each symbol represents one organoid. Data were representative of three independent experiments. Student’s *t*-test was performed to determine the statistical significance. (**P < 0.01; ***P < 0.001; ****P < 0.0001).

Given the robust phenotype implicating DNAJB9 in the ERAD of CFTR in cultured cells, we examined the role of DNAJB9 *in vivo*. CF mice (homozygous for ΔF508) are a useful model to understand the pathophysiology of disease as well as to evaluate therapies, such as an *in vivo* testing of the strategies of altering the interaction between ΔF508-CFTR and chaperone proteins^52^. Recently, primary intestinal organoids derived from mice have been successfully used by us and other groups as an important tool to test CFTR function and its interacting partners^53–55^. We hypothesized that DNAJB9 hypomorphic mice (KO) generated by gene trap technology^50^ should have increased functional CFTR on the apical membrane in the enteroids. Interestingly, DNAJB9 was highly expressed in the intestine in both WT and CF mice and expression in CF mouse intestine was comparable to WT mouse (Fig. S5). RT-PCR analysis and our previous studies^50^ show that KO mouse have minimal DNAJB9 mRNA expression and unaltered CFTR mRNA levels (Fig. S6). We first compared WT to KO mice (Fig. 3A,B). Although the extent of organoid fluid secretion was similar upon maximum forskolin (FSK) stimulation between WT and KO mice, the basal activity of CFTR was over 50% higher in KO mice. Consistently, immunoblotting of the membrane fraction from KO mouse intestine detected higher CFTR levels compared to WT (Fig. 2D), supporting the hypothesis that loss of DNAJB9 improve CFTR surface function.

Next, we asked whether DNAJB9 deficiency could rescue ΔF508-CFTR function in mice. To perform this study, DNAJB9 heterozygous (DNAJB9^+/−^) mice were first bred with CFTR^ΔF508/+^ to generate DNAJB9^+/−^CFTR^ΔF508/+^. DNAJB9^+/−^CFTR^ΔF508/+^ mice were then intercrossed to generate DNAJB9^+/+^CFTR^ΔF508/ΔF508^ and DNAJB9^−/−^CFTR^ΔF508/ΔF508^. Organoid fluid secretion assays showed that ablation of DNAJB9 increased ΔF508-CFTR activity in both resting and FSK-stimulated states (Fig. 3C,D). Taken together, these results support the hypothesis that downregulation of DNAJB9 leads to CFTR gain of function.

### DNAJB9 partial deficiency rescues ΔF508-CFTR

**Figure 4:**
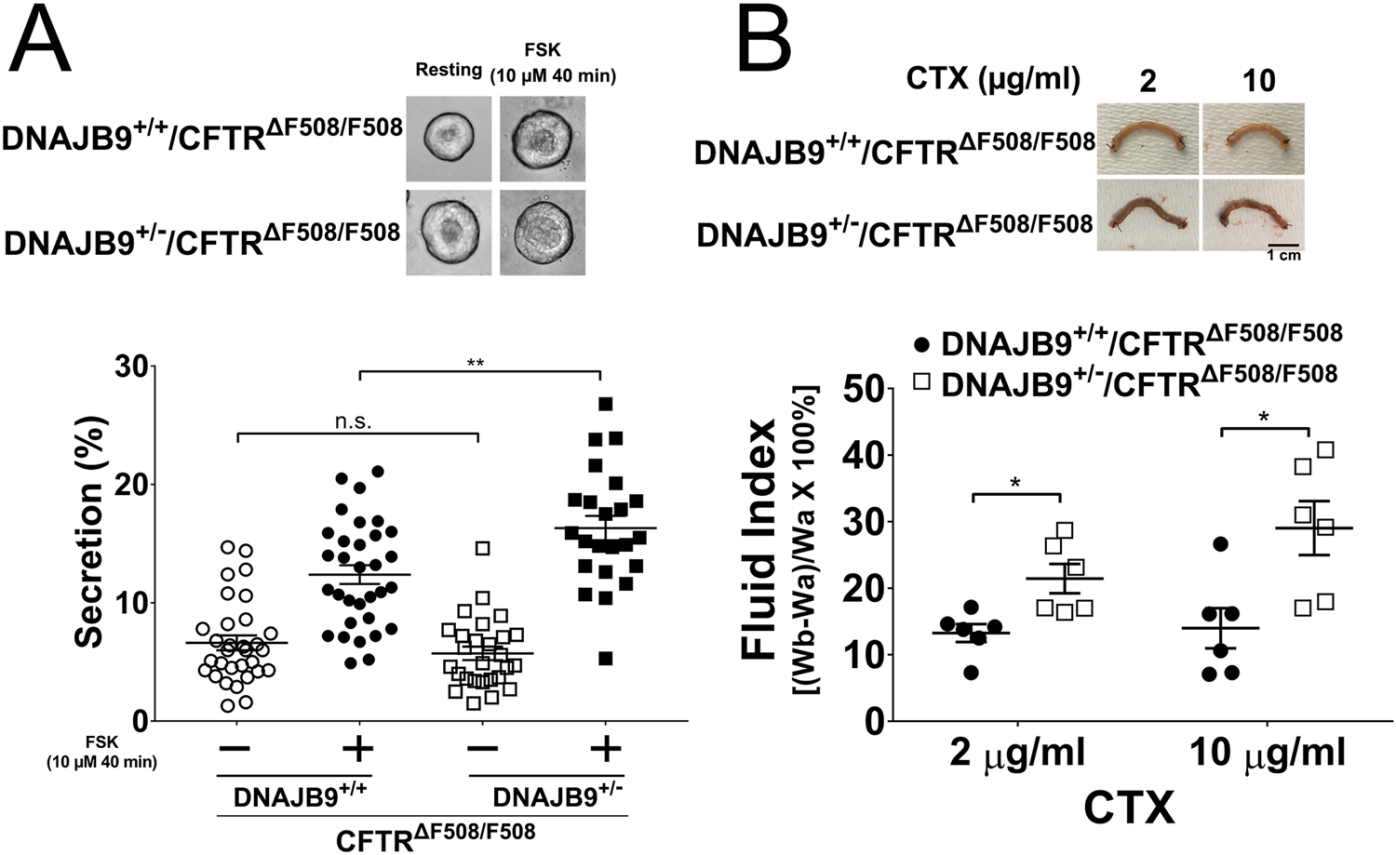
DNAJB9 may be a limiting factor for ERAD of ΔF508-CFTR. **(A)** DNAJB9 heterozygosity rescued CFTR-dependent fluid secretion in ΔF508-CFTR intestinal organoid. Intestinal organoid fluid secretion as described in “Material and Method” was performed to compare DNAJB9^+/+^CFTR^ΔF508/ΔF508^ with DNAJB9^+/−^CFTR^ΔF508/ΔF508^. Representative images before and after FSK stimulation were shown on the top. Quantification of organoid fluid secretion was shown at the bottom. Each symbol accounts for one organoid. **(B)** DNAJB9 heterozygosity enhanced CFTR-dependent cholera toxin (CTX)-induced fluid secretion in *in vivo* closed loop experiments. *In vivo* closed loop experiment was performed as described in “Materials and Methods” to compare DNAJB9^+/+^CFTR^ΔF508/ΔF508^ with DNAJB9^+/−^CFTR^ΔF508/ΔF508^. Representative images of intestinal loop were shown on the top upon two different-doses of CTX treatment. Quantification of secreted fluid were shown at the bottom. Student *t*-test was performed to determine the statistical significance. (*P < 0.05; **P < 0.01).

**Figure 5:**
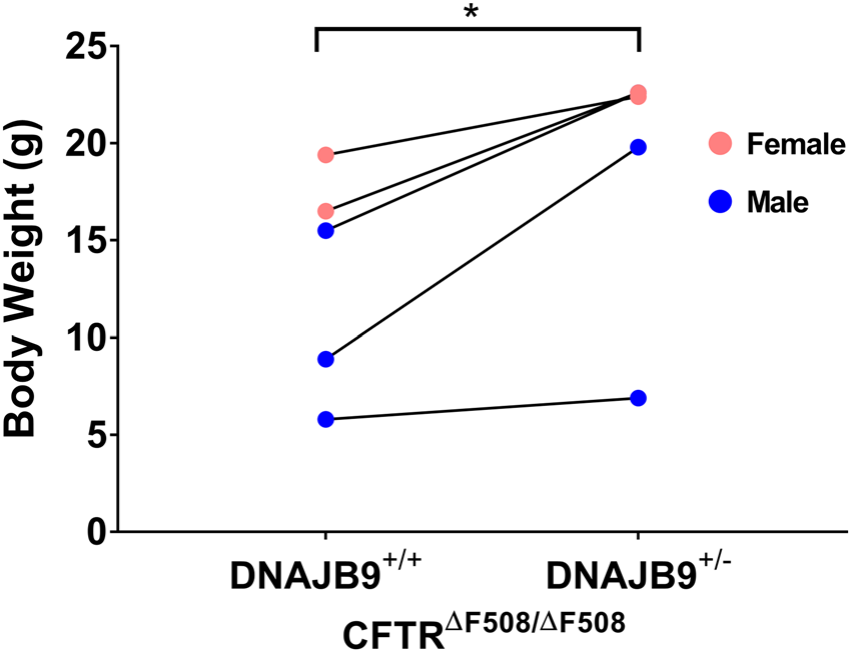
DNAJB9 heterozygosity improved ΔF508 CF mice development. Mice body weight was compared between DNAJB9^+/+^CFTR^ΔF508/ΔF508^ and DNAJB9^+/−^CFTR^ΔF508/ΔF508^ from 5 independent sibling pairs as indicated by connected line. The body weight was determined at different ages. Blue represented male and pink represent female. (*P < 0.05).

It has been observed that DNAJB9^−/−^ mice have constitutive ER stress and developmental problems^50^, and that DNAJB9^−/−^CFTR^ΔF508/ΔF508^ mice are generally smaller than their littermates DNAJB9^+/+^CFTR^ΔF508/ΔF508^. However, DNAJB9 heterozygous mice are asymptomatic^50^ and in HEK293 cells 30% reduction of DNAJB9 could rescue ΔF508-CFTR (Fig. 2); therefore, we rationalized that partial knockdown of DNAJB9 in DNAJB9 heterozygous (DNAJB9^+/−^) is likely to rescue ΔF508-CFTR. As shown in Fig. 4A, compared to DNAJB9^+/+^CFTR^ΔF508/ΔF508^ mice, DNAJB9^+/−^CFTR^ΔF508/ΔF508^ mice had increased organoid fluid secretion by ~31%. To further assess this “rescue” effect *in vivo*, intestinal closed-loop experiments were performed, which assess CFTR function of the intact gut as previously described^54,56^. In contrast to organoid fluid secretion assay where FSK was used to activate CFTR, cholera toxin (CTX) was used to stimulate CFTR in the closed-loop assay. DNAJB9 heterozygosity upregulated CFTR-dependent fluid secretion by ~60% and ~107% following 2 µg/mL and 10 µg/mL CTX treatment, respectively (Fig. 4B), consistent with an increase in functional ΔF508-CFTR when DNAJB9 gene dose is decreased. Lastly, we examined whether heterozygous DNAJB9 could improve the CF mice development by measuring their body weight since CF mice usually have reduced body weight because of lack of CFTR function^51^. It is found that in a sibling pair of same sex, CF mice with DNAJB9 heterozygosity had increased body weight compared to CF mice (Fig. 5), which is not dependent on sex and age.

## Discussion

Endoplasmic Reticulum-Associated Degradation (ERAD) pathways have been widely appreciated to play a major role in the removal of “misfolded” CFTR from ER. Using different approaches, many important players have been identified, including cytosolic chaperones and ER membrane proteins. Increasing evidence suggests that ER luminal factors are also needed to mediate ERAD of misfolded proteins, including transmembrane protein opsin, mutation of which is associated with autosomal dominant retinitis pigmentosa^57,58^. However, the luminal factor(s) that participate in CFTR ERAD are still completely unknown. It may be possible that the involvement of ER luminal factors in CFTR ERAD has been simply neglected because (1) majority of the CFTR mutations occur in the cytosolic or membrane domains (www.cftr2.org), (2) the luminal loops or extracellular loops (ECLs) of CFTR are small, accounting for only 7% CFTR mass with the biggest loop, ECL4, harboring only 39 amino acids, and (3) the cytosolic or membrane factors appear to have enough “power” to mediate CFTR ERAD. It might be also due to lack of tools, such as antibodies, to identify these factors. Using purified full-length active CFTR coupled with ProtoArray, we have shown here that protoArray had distinct ability to identify CFTR-interacting partners in CFTR structure of various cellular compartments, including the ECLs of CFTR. It is found that DNAJB9 interacted with CFTR with relatively high signal intensity (Fig. 1C). Overexpression of DNAJB9 increased CFTR ubiquitination (Fig. S4). Knockdown DNAJB9 increased CFTR surface and functional expression *in vitro* and *in vivo* (Figs 2–4). These evidences suggest that DNAJB9 regulates the CFTR ERAD pathway and that interrupting DNAJB9 function may be a novel strategy to rescue mutant CFTR because heterozygosity of DNAJB9 improve overall CF mice development (Fig. 5).

Interestingly, in an earlier microarray gene analysis^59^, gene expression profile of ΔF508 homozygous CF patients was compared between patients with mild lung disease and patients with severe lung disease. It is found that DNAJB9 was one of the highly expressed genes in human nasal epithelial cells, and compared to patients with mild lung disease, patients with severe lung disease were associated with higher DNAJB9 expression (Fig. S7). This evidence further supports that targeting DNAJB9 could be an effective strategy for CF treatment.

DNAJB9 is a unique ER luminal co-chaperone^35^. Our data indicates that DNAJB9 interacts directly with CFTR. However, it is unclear exactly how CFTR interacts with this co-chaperone. DNAJB9 belongs to type II DNAJ (Hsp40) homology subfamily B, containing an N-terminal J-domain which was suggested to regulate nucleotide binding cycles of Hsp70, a G/F domain in the middle, and C-terminal domain (CTD) which was shown to interact with its substrate^47^. Therefore, it is possible that it is through its CTD that DNAJB9 interacts with CFTR. However, we do not rule out the possibility that their interaction is indirect (i.e., via an intermediary partner), which would result in the same outcome. More experiments are needed to determine this and is beyond the scope of this study.

Recently, it has been elegantly demonstrated that DNAJB9 prefers binding to aggregation-prone polypeptide in a BiP-independent manner and mediates the substrate to ubiquitination-proteasomal degradation pathway^37^. Interestingly, when CFTR ECL4 sequence was subjected to TANGO algorithm analysis which has been used by Behnke *et al*. to identify aggregation-prone region in their designed peptide library^37^, it was found that CFTR ECL4 has one aggregation-prone region at each end of the loop (Fig. S8). Particularly, a region at the C-terminal of ECL4 has great potential to form aggregates. Therefore, we propose that DNAJB9 via its CTD domain interacts with CFTR via ECL4. Interestingly, it seemed that DNAJB9 has no preference to either WT-CFTR or ΔF508-CFTR (Fig. 1). Importantly, deficiency of DNAJB9 could rescue both WT- and ΔF508-CFTR. These data suggest that the interacting region in both WT-CFTR and ΔF508-CFTR have equal accessibility to DNAJB9.

It has been widely accepted that DNAJB9 could mediate ERAD of its clients^36,37^, however, the molecular mechanism is unclear. Previous studies have suggested that interrupting ERAD-related (co-)chaperone function by siRNA could rescue ΔF508-CFTR^14,51^. This effect was also observed in our studies of DNAJB9, using both siRNA studies (Fig. 2) and a genetic ablation animal model (Figs 3–5), suggesting a critical role of DNAJB9 in CFTR ERAD pathway. Importantly, DNAJB9 is a soluble ER luminal protein^35^, distinct from previous studies focusing on cytosolic regulators, suggesting that ER luminal factors indeed participate in ERAD of misfolded integral membrane proteins, including CFTR. But, how does DNAJB9 execute its role? It is noteworthy that BiP protein is not required for DNAJB9-client interaction^36,37^ and that BiP is not associated with CFTR complex^26–28^, suggesting that DNAJB9-mediated ERAD of CFTR is likely independent of BiP. It has been shown that multiple E3 ligases on the ER-membrane are involved in CFTR ubiquitination for degradation^14,51,60,61^. It is possible that DNAJB9 plays a role in directing misfolded CFTR to one of these E3 ligases and thus promoting CFTR ubiquitination because overexpressing DNAJB9 increased ubiquitination of CFTR at steady state (Fig. S4). Therefore, it would be interesting to determine which E3 ligase is involved in this pathway. Given the fact that DNAJB9 is not a membrane anchored protein, one could postulate that there must be an adaptor protein linking DNAJB9 to the membrane E3 ligase. Therefore, it is equally important to identify any potential adaptor protein which delivers DNAJB9-substrate complex to E3 ligase. Lastly, our siRNA study (Figs 2 and S3) and animal heterozygosity (Figs 4 and S6), where DNAJB9 mRNA were only partially downregulated, suggesting that DNAJB9 might be a limiting factor in CFTR ERAD pathway and that targeting DNAJB9 using specific inhibitor may turn out to be an important strategy in correcting Cystic Fibrosis effectively.

## Materials and Methods

### Chemical and antibodies

Chemical used in this study included Forskolin, CTX were from MilliporeSigma. SPQ [6-methoxy-N-(3-sulfopropyl)quinolinium], BSA (bovine serum albumin) were from ThermoFisher.

The antibodies used in this study included anti-CFTR antibody (596) from CF foundation antibodies distribution program at UNC Chapel Hill; anti-CFTR [clone 1314,^62^], homemade; anti-HA (3724), anti-vinculin (13901), anti-FLAG (14793), anti-GAPDH (2118), anti-ubiquitin (3936), from Cell Signaling; Anti-Na/K ATPase (sc-28800) from Santa Cruz. Anti-Rabbit-HRP, anti-Mouse-HRP and anti-DNAJB9 (HPA041553) were from MilliporeSigma.

### Cell transfection

HEK293 cells were obtained from ATCC and were maintained in DMEM/F-12 medium containing 10% FBS and 1% Penicillin-Streptomycin. To do vector transfection, cells were transfected with human DNAJB9-HA [3HA was expressed at the very C-terminal of the protein^36^] using Lipofectamine 3000 (ThermoFisher Scientific) according to the manufacturer’s protocol. To do siRNA transfection, cells were transfected with DNAJB9 or RNF5 [HSS155077, ThermoFisher] specific siRNA by using RNAiMax (ThermoFisher) according to the manufacturer’s protocol. Transfected cells were studied 36–48 hours post-transfection.

### Mouse jejunum membrane fraction preparation

Mouse Jejunum in sucrose buffer (sucrose 250 mM, Tris-HCl 10 mM, EDTA 1 mM, pH 7.2) were homogenized in a tissue grinder on ice in the presence of protease inhibitors (PMSF, Leupeptin, APR). After brief centrifuge at 800 × g for 10 min at 4 °C, the supernatant was centrifuged at 200,000 × g for 60 min at 4 °C. The pelleted membrane fraction was used for immunoblotting.

### Cell Lysate preparation and immunoprecipitation

HEK293 cells stably express FLAG-ΔF508-CFTR and FLAG-WT-CFTR were lysed in IP buffer (87787, ThermoFisher) containing EDTA-free protease-inhibitor cocktail (MilliporeSigma). Cell debris were removed by centrifugation at max speed for 10 min at 4 °C. FLAG-CFTR and DNAJB9-HA were immuneprecipitated from whole-cell lysates using anti-FLAG-conjugated resin (MilliporeSigma) and anti-HA-conjugated resin (MilliporeSigma), respectively. Proteins immobilized on beads were eluted by SDS-PAGE Sample Buffer. Samples were incubated for 20 min at 37 °C before subjected to SDS-PAGE and western blotting following standard protocols. Images were acquired using Bio-Rad ChemiDoc^TM^ Touch imaging System, processed using Image Lab (version 6.0), and collected in Photoshop (version 19.1.6).

### Real time PCR

RNA from HEK293 cells and from mouse ileum were prepared by using Qiagen RNeasy Mini Kit and TRIZOL reagent (ThermoFisher) according to manufacturer’s protocols. RNA SuperScript III Reverse Transcriptase (ThermoFisher) kit was used to synthesize cDNA. SYBR Green-based Real time PCR was performed using pre-validated primers for human and mouse CFTR, DNAJB9, 18 S genes. Assay was performed using QuantStudio^TM^ 5 (Thermo) and analyzed by StepOne software (version 2.3).

### DSP crosslinking

After washed with once PBS, cells were subjected to chemical cross-linking at RT for 30 min using 1 mM DSP [dithiobis(succinimdylproprionate), ThermoFisher]. The reaction was quenched by 1 M Tris (pH 7.5) at RT for 15 min.

### Full length FLAG-CFTR purification and activity measurement

HEK293 cells expressing full-length FLAG-CFTR were lysed using lysis buffer (0.2% Triton X-100 in PBS) in the presence of protease inhibitors and cells debris was removed by centrifugation^63^. Whole cell lysates (or purified microsomes) were subjected to anti-FLAG-conjugated resin column. After extensive washing, bound proteins were eluted with elution buffer (100 mM Glycine, pH 2.2) containing 0.2% Triton X-100 and then neutralized with 1 M Tris. Purified protein mixture were dialyzed with dialysis buffer. The purity were determined by SDS-PAGE followed by Coomassie Blue staining and quantitated by densitometry analysis. The ATPase activity of purified CFTR was measured using radiolabeled [γ-^32^P]ATP by incubation for the indicated time followed by Thin-layer Chromatography (TLC).

### ProtoArray

ProtoArray (ThermoFisher) was performed using the above purified full-length FLAG-CFTR according to manufacturer’s protocol.

### Tango analysis

Tango analysis was done using the online tool at http://tango.switchlab.org/ for prediction of aggregating regions in unfolded polypeptide chains.

### WES assay

Total cell lysate or IP samples were prepared in lysis buffer (RIPA from Cell Signaling or IP lysis buffer from ThermoFisher) containing protease inhibitor cocktail, and then subjected to WES system according to manufacturer’s protocol using the indicated antibodies. Data were analyzed using Compass for SW version 3.1.7.

### SPQ assay

HEK293 cells stably expressing WT-CFTR and ΔF508-CFTR were transfected with DNAJB9-specific siRNA [HSS106410, ThermoFisher,^36^]. Cell were plated in a 96 well plate with clear bottom and black well plate 24 hours after transfection. After overnight incubation, cells were acutely loaded with 10 mM SPQ prepared in 1:1 Opti-MEM:water for 5 min at 37 °C. After aspirating SPQ, cells were washed once with NaI buffer [130 mM NaI, 20 mM HEPES, 10 mM glucose, 4 mM KNO_3_, 1 mM Ca(NO_3_)_2_∙H_2_O, and 1 mM Mg(NO_3_)_2_], and then continue incubation at room temperature (RT) in NaI buffer for 1 hour with one time replacement with fresh NaI buffer. Cells were then washed once with NaNO_3_ buffer [130 mM NaNO_3_, 20 mM HEPES, 10 mM glucose, 4 mM KNO_3_, 1 mM Ca(NO_3_)_2_∙H_2_O, and 1 mM Mg(NO_3_)_2_] and then 100 µL NaNO_3_ buffer was added to each well and SPQ signal were measured at 360 EX/460 EM for 5 minutes using Flex-station3 (Molecular Devices) to record the baseline. To measure SPQ signal at FSK stimulated phase, additional 100 µL NaNO_3_ buffer containing 10 µM FSK was added to each well. Lastly, additional 100 µL NaI buffer was added to quench the SPQ signal. Signal from each well was normalized to signal at 5^th^ min.

### In-cell western (ICW)

HEK293 cells 24 hours after transfection were plated on 96 well plate and fixed with 4% formaldehyde for 15 min at RT. After washing with PBS, cells were incubated with blocking buffer (10% BSA in PBS) for 1 hour at RT and then with anti-FLAG antibody in 3% BSA in PBS for overnight at 4 °C. After thorough washing, cells were incubated with IRDye® 800CW-conjugated anti-Rabbit antibody (ProteinSimple) containing CellTag 700 (ProteinSimple) for 1 hour. After washing, buffer was aspirated from each well and the plate was scanned and quantitated using LI-COR Odyssey®CLx imaging system.

### Proximity ligation assay (PLA) with T84 cells

T84 cells grown on glass cover slips were fixed with 4% formaldehyde for 15 min at RT and permeabilized by 0.3% Triton-100 for 15 min at 37 °C. Cells were then blocked in 2.5% goat serum for 2 hr at RT. Cells were incubated with mouse anti-CFTR antibody in the presence or absence of rabbit anti-DNAJB9 antibody at 4 °C overnight. For the proximity ligation assay, anti-rabbit (plus) and anti-mouse (minus) Duo link *In Situ* PLA probes (Millipore, Sigma) were added to the samples. The next steps of PLA assay were completed as described in the manufacturer’s protocol. Slides were examined using a confocal microscope (Olympus FV1200).

### Mice

CFTR^ΔF508/ΔF508^ mice (CF mice) used in this study were originally from Kirk R Thomas at the University of Utah (Salt Lake City, Utah, USA)^52^. DNAJB9 mice were as described before^50^. All mice are maintained in the barrier facility at CCHMC and were fed with normal chow and regular water except the mice harboring CFTR^ΔF508/ΔF508^ that were treated with Colyte water containing osmotic laxative composed of Polyethylene Glycol 3350 (18 mM), NaCl (25 mM), KCl (1 mM), NaHCO_3_ (20 mM) and anhydrous Na_2_SO_4_ (40 mM).

### Intestinal crypt isolation and quantitation of fluid secretion in organoids

Mouse intestinal crypt preparation and fluid secretion quantitation have been described previously^55^. It is noted that fluid secretion were stimulated by forskolin for the indicated time as shown in figures.

### Intestinal fluid secretion (*in vivo*) measurement or closed intestinal loop experiment

The procedure was performed as described previously^54^. Briefly, mice that were subjected to this procedure were 10–20 weeks old and were deprived of food for 24 hours prior to surgery. Mice were then anesthetized using isoflurane and physiological conditions (O_2_ and body temperature) were maintained through whole procedure. The distal region of the small intestine were exposed by making a small abdominal incision. Intestinal loops (~2 cm) were made in such a way that adjacent loops were separated by around 1 cm. The closed loop were then injected with 100 µL of PBS containing 2 µg/mL or 10 µg/mL CTX. The incisions on abdomen and skin were closed with surgical sutures. The mice were allowed to recover for 6 hours. At the end of recovery, the mice were sacrificed by CO_2_. Intestinal loops were harvested, and weighted before (F1) and after (F2) the fluid inside the loop was absorbed. Fluid secretions were quantitated by measuring the value of [(F1-F2)/F2].

### Statistics

The statistical significance was performed using Student’s-*t* test in GraphPad version 7.0.

### Study approval

All mouse procedures in this study were performed under protocols approved by Cincinnati Children’s Hospital Medical Center’s Institutional Animal Care and Use Committee, and in compliance with institutional and regulatory guideline.

## Supplementary information

Dataset 1

## Data Availability

The datasets generated during and/or analyzed during the current study are available from the corresponding author on reasonable request.
