# Supplementary material for: Targeting DNAJB9, a novel ER luminal co-chaperone, to rescue ΔF508-CFTR
Source: Sci Rep. 2019 Jul 8;9:9808. doi: 10.1038/s41598-019-46161-4 (PMC6614449; doi:10.1038/s41598-019-46161-4)
Supplement: Supplementary file 1 — Dataset 1 [file 41598_2019_46161_MOESM1_ESM.pdf]

Title: Targeting DNAJB9, a novel ER luminal co-chaperone, to rescue ΔF508-CFTR

Authors: Yunjie Huang[1], Kavisha Arora[1], Kyu-Shik Mun[1], Fanmuyi Yang[1], ChangSuk Moon[1], Sunitha Yarlagadda[1], Anil Jegga[2], Timothy Weaver[3], and Anjaparavanda P. Naren[1]*

Affiliations:
[1]Division of Pulmonary Medicine, Cincinnati Children's Hospital Medical Center, Cincinnati, OH 45229.
[2]Division of Biomedical Informatics, Cincinnati Children's Hospital Medical Center, Cincinnati, OH 45229.
[3]Division of Pulmonary Biology, Cincinnati Children's Hospital Medical Center, Cincinnati, OH 45229.

*To whom correspondence should be addressed:
Anjaparavanda P. Naren
Cincinnati Children's Hospital Medical Center
R4041
3333 Burnet Ave.
Cincinnati, OH 45229
Phone: 513-636-3796
Email: anaren@cchmc.org

Fig S1

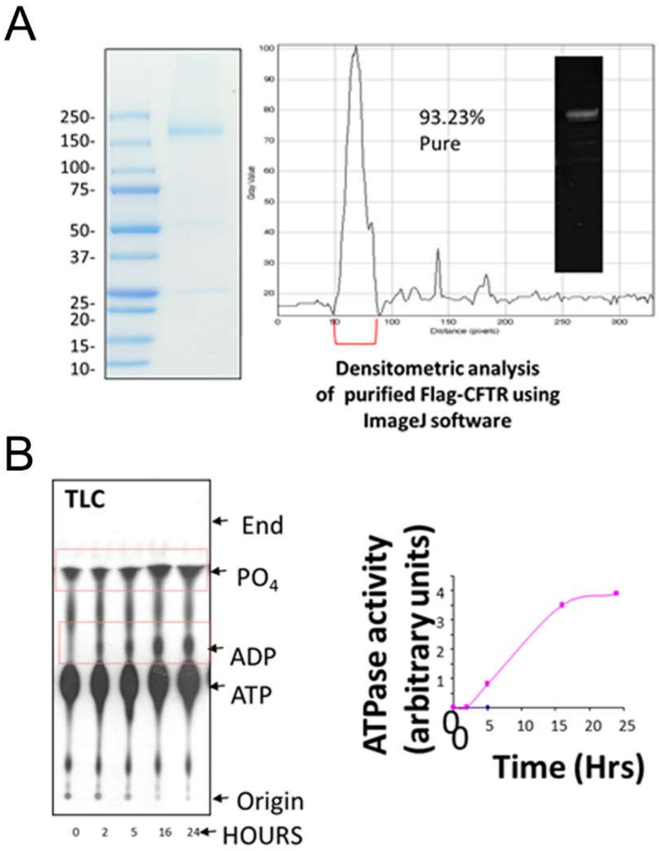

Figure S1. Purified FLAG-CFTR was active. **(A)** Coomassie Blue staining of purified FLAG-CFTR and its purity was quantified by densitometry analysis using image J. **(B)** The ATPase activity of purified FLAG-CFTR was determined by incubating FLAG-CFTR with [γ-^{32}P]ATP for indicated time followed by thin-layer-chromatography (TLC). Left is the representative image of TLC and right is the quantification of ADP, an index of ATPase activity, over the time.

Fig S2

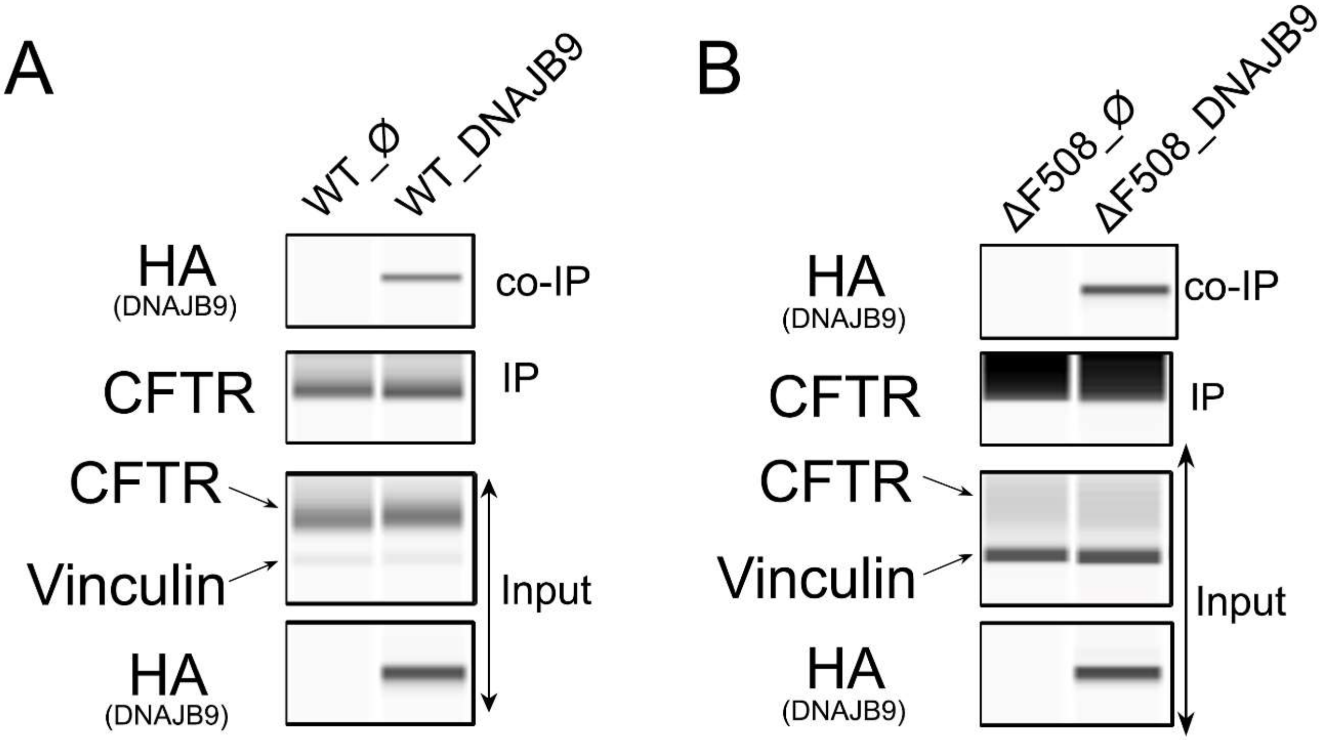

Figure S2. DNAJB9 is associated with CFTR in cells. WES data of co-immunoprecipitation. HEK293 cell lines stably expressing WT- **(A)** or ΔF508- **(B)** CFTR were transiently transfected with empty vector (Ø) or DNAJB9-HA (DNAJB9). After 48 hours, cells were treated with crosslinker DSP at RT, followed by quenching with Tris. Cell lysates were then subjected to immunoprecipitation using anti-FLAG M2 beads in the absence of any reducing agent. Proteins in the pellet were eluted in the lysis buffer containing DTT. Total cell lysate (input) and precipitate were subjected to WES system using anti-CFTR, anti-vinculin and anti-HA antibodies.. Data was representative of three independent experiments.

Fig S3

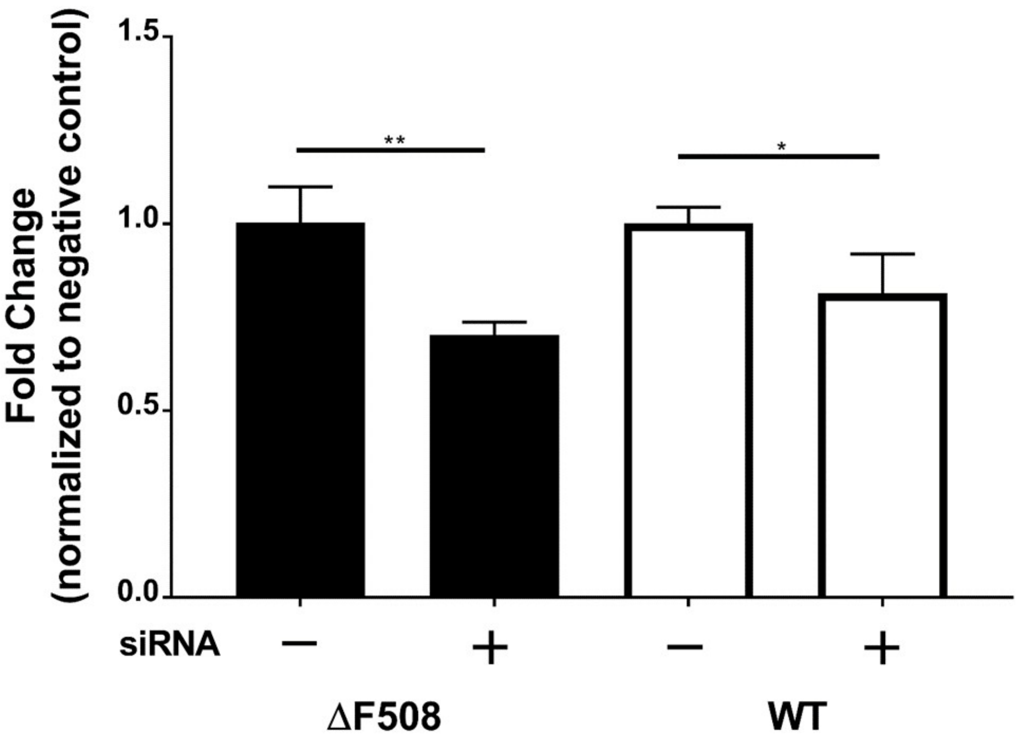

Figure S3. DNAJB9 mRNA was knockdown by siRNA treatment. HEK293 cell lines stably expressing WT- or ΔF508-CFTR were transiently transfected with empty vector (-) or DNAJB9 siRNA (+) for 48 hours. RNA was isolated using Qiagen Kit, and RT-PCR was performed to determine DNAJB9 levels. 18S was used as internal control. Data was representative of three independent experiments. Student *t*-test was performed to determine the statistical significance. (*P<0.05; **P<0.01).

Fig S4

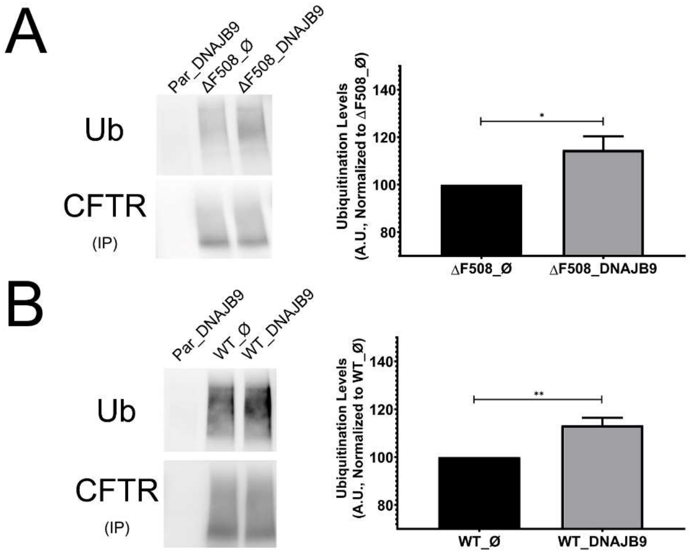

Figure S4. Overexpression of DNAJB9 enhanced CFTR ubiquitination. HEK293 cell lines that stably expressed ΔF508- (A) and WT- (B) CFTR were transiently transfected with empty vector (Ø) or DNAJB9 vector (DNAJB9). Parental HEK293 cells that was transiently transfected with DNAJB9 was included as specificity control. CFTR was immunoprecipitated by anti-FLAG antibody 48 hours post-transfection. Ubiquitin and CFTR were probed using anti-ubiquitin and anti-CFTR antibodies, respectively. Data was representative of three independent experiments. Student *t*-test was performed to determine the statistical significance. (**P*<0.05; ***P*<0.01).

Fig S5

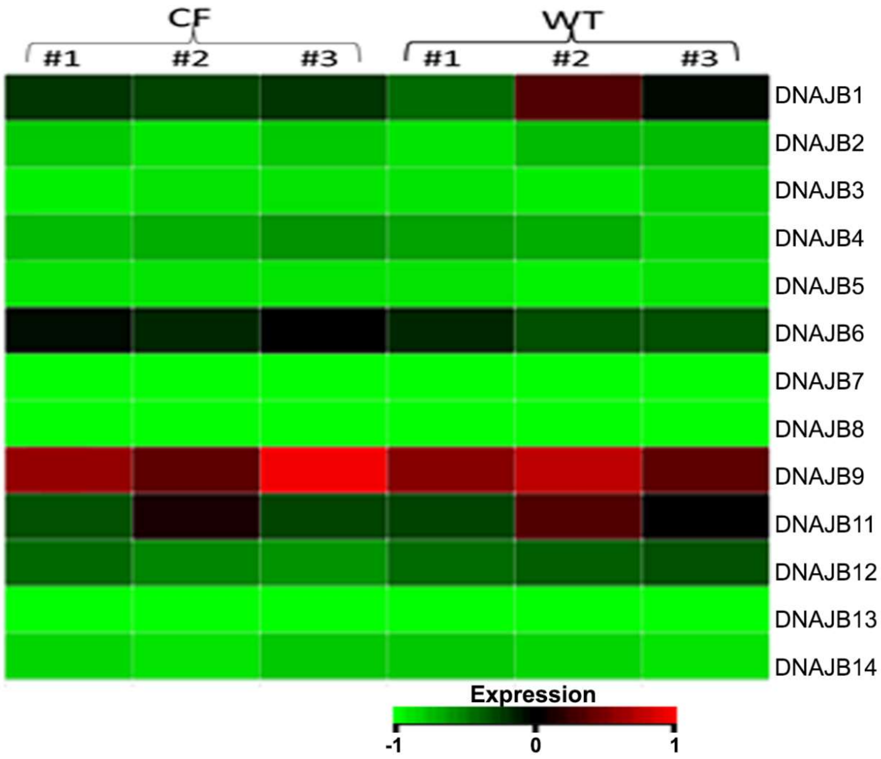

Figure S5. DNAJB9 is highly expressed in mouse intestine. Heat map of small intestine gene expression profile of WT and CF mice was determined by RNAseq.

Fig S6

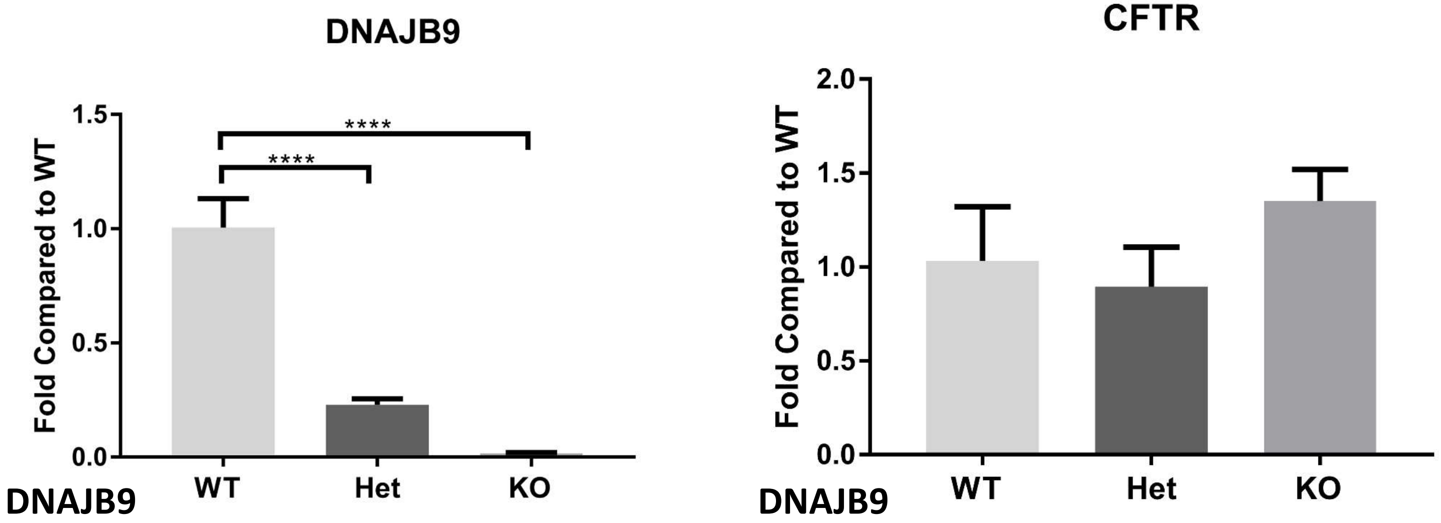

Figure S6. Genetic knockdown of DNAJB9 didn't alter CFTR expression. RT-PCR analysis of DNAJB9 and CFTR in Mouse ileum Tissue from different genotypes, DNAJB9$^{+/+}$ (WT), DNAJB9$^{+/-}$ (Het), and DNAJB9$^{-/-}$ (KO).

Fig S7

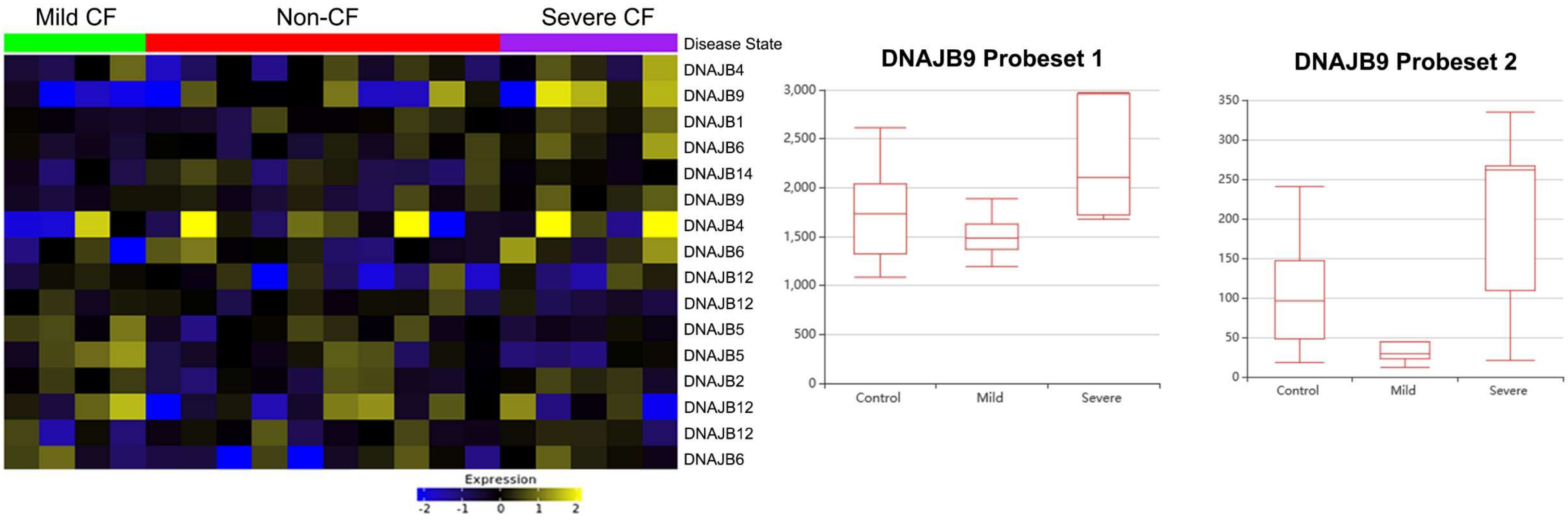

Figure S7. DNAJB9 is highly expressed in human nasal respiratory epithelial cells. Data mining from study (Wright, JM *et al.*, Am J Respir Cell Mol Biol. 2006 Sep;35(3):327-36) in which gene expression profile of nasal epithelial cells from non-CF, mild-CF, and severe CF was determined. (Left) Heat map of gene expression of DNAJB (Hsp40) family. (Right) DNAJB9 expression in both probesets on the microarray is higher in severe CF patients than in mild CF patients.

Fig S8

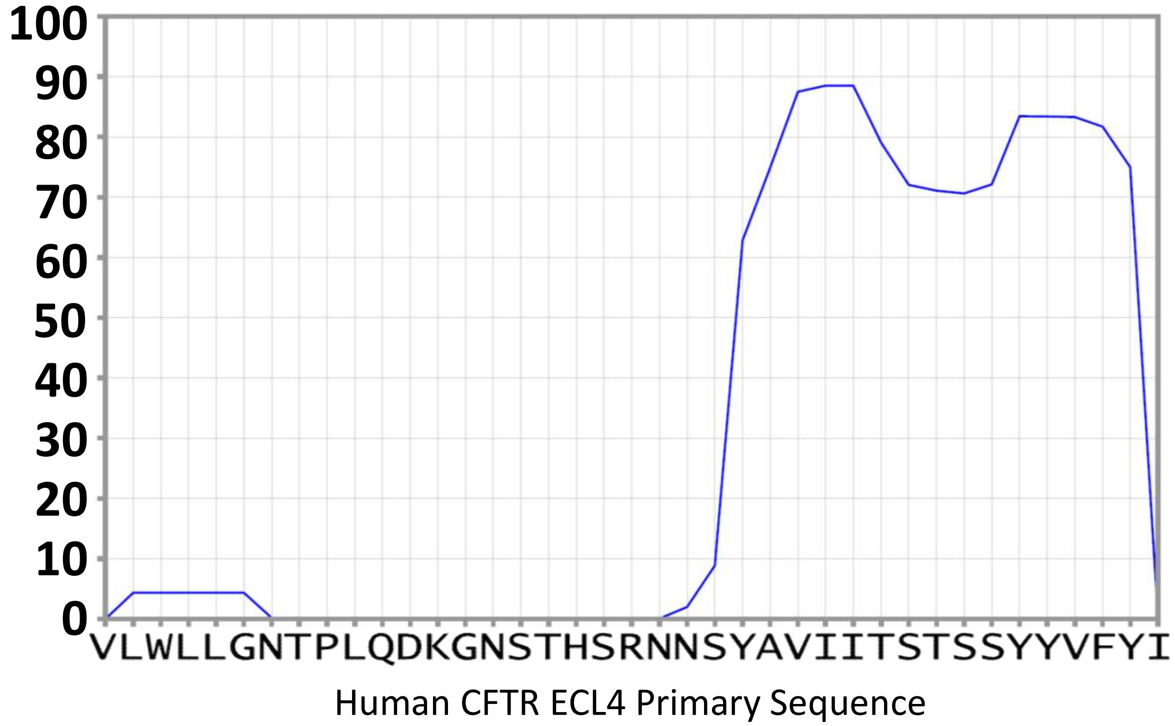

Figure S8. Tango Plot of human CFTR Extracellular Loop 4 (ECL4). The higher value indicated higher aggregation potency.

Fig S9

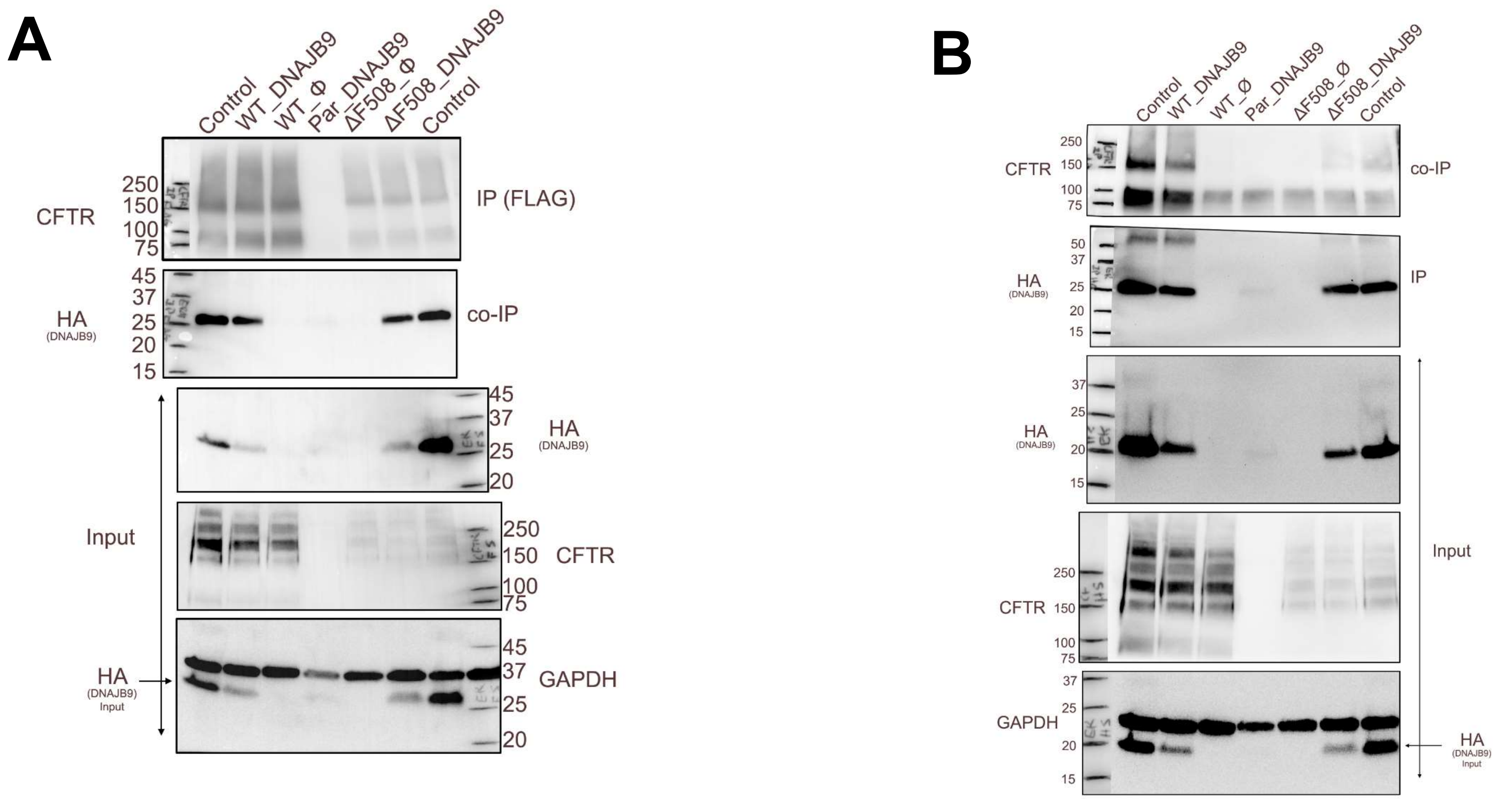

Figure S9. Full-length blots of Figure 1D and 1E. Wester blotting of co-immunoprecipitation. HEK293 cell lines stably expressing WT- and ΔF508-CFTR were transiently transfected with empty vector (Ø) or DNAJB9-3HA vector (DNAJB9). Parental HEK293 cells transfected with DNAJB9-3HA were used as specificity control. Forty-eight hours after transfection, immunoprecipitation was performed using anti-FLAG antibody beads (A) and anti-HA antibody beads (B). Precipitate and total cell lysate (input) were then subjected to Western Blotting using anti-CFTR, anti-HA and anti-GAPDH antibodies. Data were representative of three independent experiments.

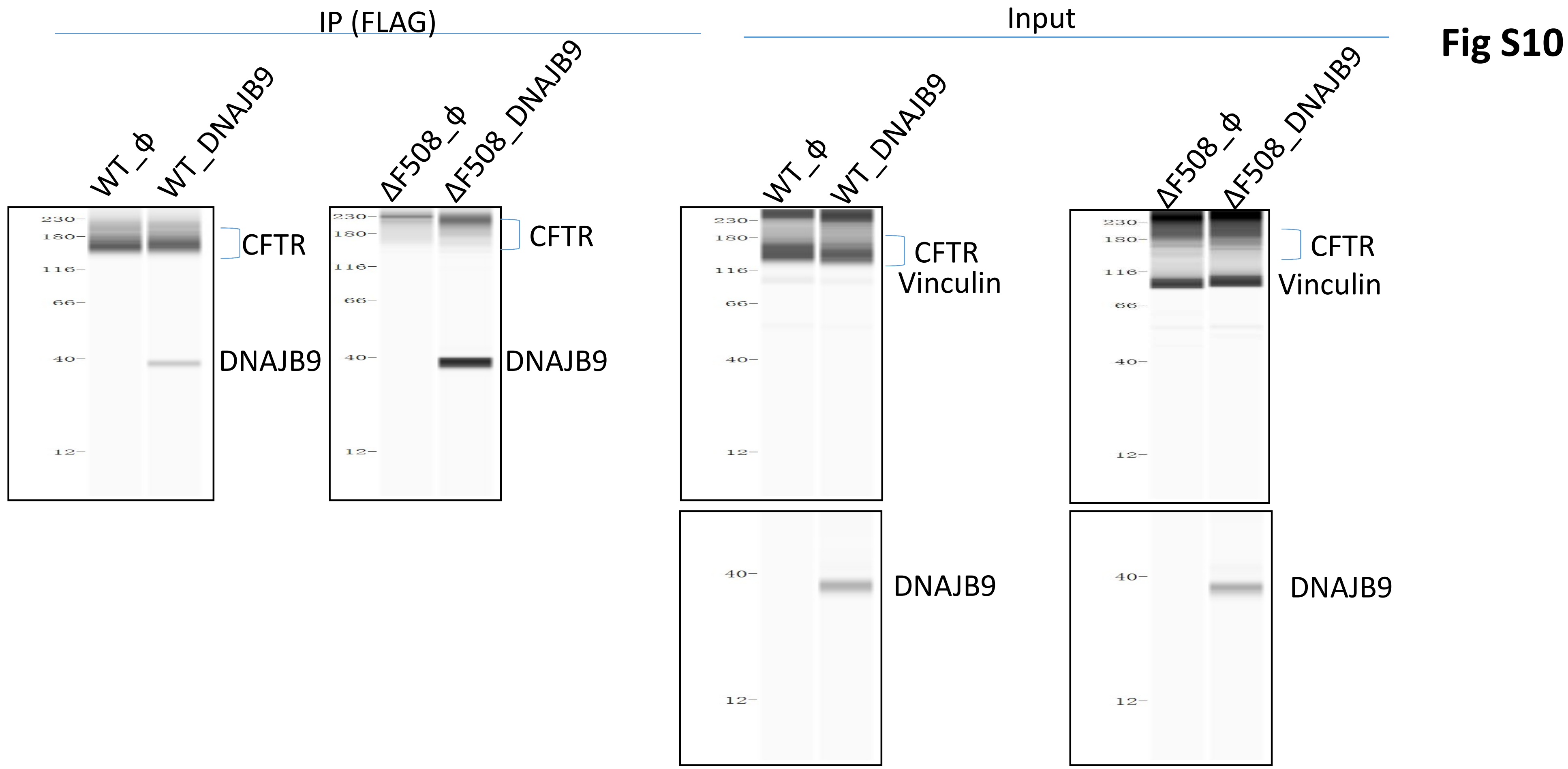

Figure S10. Full-length blots of Figure S2. DNAJB9 is associated with CFTR in cells. WES data of co-immunoprecipitation. HEK293 cell lines stably expressing WT- or ΔF508-CFTR were transiently transfected with empty vector (Ø) or DNAJB9-HA (DNAJB9). After 48 hours, cells were treated with crosslinker DSP at RT, followed by quenching with Tris. Cell lysates were then subjected to immunoprecipitation using anti-FLAG M2 beads in the absence of any reducing agent. Proteins in the pellet were eluted in the lysis buffer containing DTT. Total cell lysate (input) and precipitate were subjected to WES system using anti-CFTR, anti-vinculin and anti-HA antibodies. Data was representative of three independent experiments

Fig S11

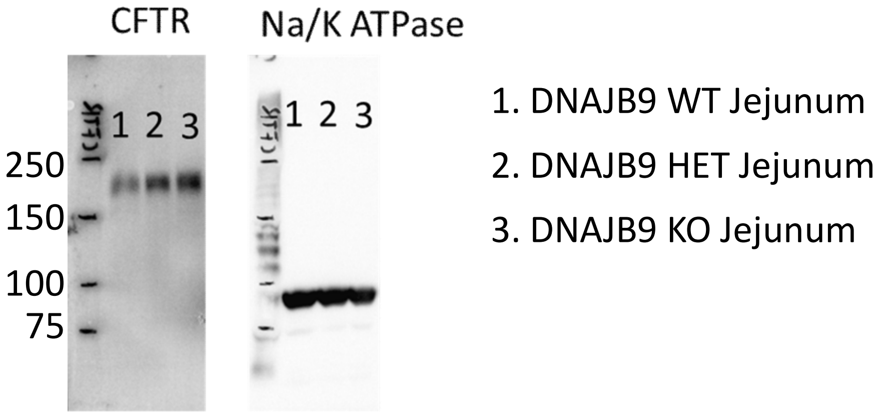

Figure S11 Knockdown DNAJB9 increased CFTR expression. Immunoblotting of mouse Jejunum membrane fraction from different genotypes, DNAJB9$^{+/+}$ (WT), DNAJB9$^{+/-}$ (Het), and DNAJB9$^{-/-}$ (KO). CFTR and Na/K ATPase was probed using anti-CFTR and anti-Na/K ATPase antibodies, and Na/K ATPase was served as a membrane marker and loading control.
